# Professional Skills and Competence for Safe and Effective Procedural Sedation in Children: Recommendations Based on a Systematic Review of the Literature

**DOI:** 10.1155/2010/934298

**Published:** 2010-06-28

**Authors:** Piet L. J. M. Leroy, Daphne M. Schipper, Hans (J. ) T. A. Knape

**Affiliations:** ^1^Pediatric Sedation Unit, Department of Pediatrics, Maastricht University Medical Centre, P.O. Box 5800, 6202 AZ Maastricht, The Netherlands; ^2^Dutch Institute for Health Care Improvement CBO, P.O. Box 20064, 3502 LB Utrecht, The Netherlands; ^3^Department of Anesthesiology, University Medical Centre, P.O. Box 85500, 3508 GA Utrecht, The Netherlands

## Abstract

*Objectives*. To investigate which skills and competence are imperative to assure optimal effectiveness and safety of procedural sedation (PS) in children and to analyze the underlying levels of evidence. *Study Design and methods*. Systematic review of literature published between 1993 and March 2009. Selected papers were classified according to their methodological quality and summarized in evidence-based conclusions. Next, conclusions were used to formulate recommendations. *Results*. Although the safety profiles vary among PS drugs, the possibility of potentially serious adverse events and the predictability of depth and duration of sedation define the imperative skills and competence necessary for a timely recognition and appropriate management. The level of effectiveness is mainly determined by the ability to apply titratable PS, including deep sedation using short-acting anesthetics for invasive procedures and nitrous oxide for minor painful procedures, and the implementation of non-pharmacological techniques. *Conclusions*. PS related safety and effectiveness are determined by the circumstances and professional skills rather than by specific pharmacologic characteristics. Evidence based recommendations regarding necessary skills and competence should be used to set up training programs and to define which professionals can and cannot be credentialed for PS in children.

## 1. Introduction

Invasive diagnostic procedures are a part of daily pediatric practice. Many of these procedures are painful, stressful, and impossible to perform without immobilizing the patient. Therefore, procedural sedation (PS) is required to enable these procedures to be performed. PS can be defined as the use of sedative, analgesic, or dissociative drugs in order to provide anxiolysis, analgesia, sedation, and motor control during painful or unpleasant procedures [1].

Since anesthesiologists cannot cover the growing demand for PS, nonanesthesiologists have organized their own PS strategies [2, 3]. Historically, this resulted in a wide range of drugs and techniques for use in pediatric PS, involving a large variance of sedation levels, sedation level predictability, effectiveness, and associated risks. However, by the end of last century, PS by nonanesthesiologists was increasingly criticized by anesthesiologists for neglecting transparency and standard safety precautions. There are strong indications that within this criticism, a source could be found for PS-related accidents [4, 5]. About a decade ago, dedicated nonanesthesiology specialists, who recognized the urgent need to improve the safety and quality of PS in children, joined the initial criticism by anesthesiologists, pointing at the safety problems of PS by the untrained. In order to prevent PS-related tragedies, guidelines on PS were published [1, 6–10]. In summary, these guidelines specify safety precautions that include the assessment of the risk of sedation prior to PS, informed consent, guidelines on proper fasting status, appropriate monitoring, recovery standards, appropriate rescue facilities, and specific professional skills and competence. Generally recommended skills and competence are: the ability to perform a preprocedural risk analysis, practical knowledge and experience of applied sedatives, the ability to implement the necessary monitoring and surveillance, the ability to recognize and interpret sedation levels, and the ability to immediately recognize and adequately treat any unwanted side effects or complications, particularly hypoventilation and airway obstruction. These recommendations are mainly based on indirect evidence, expert opinion, “common sense”, and widely accepted safety rules for general anesthesia. The adoption of a uniform and systematic practice is associated with a significant reduction in adverse events during anesthesia [11]. Similarly, there is strong evidence that implementation of published guidelines leads to safer and more effective PS [12–15].

However, despite the availability of guidelines, PS practice is still unsafe in many settings and adherence to guidelines among nonanesthesiologists has been reported to be low [16, 17]. It has been argued that guidelines on PS produced by consensus between anesthesiologists (rather than on evidence-based guidelines by the clinicians themselves) have caused confusion and variation in practices [3].

Recent papers focus increasingly on the duty to deliver effective PS, not only from a procedural point of view (i.e., guaranteeing predictable procedural success and timing) but also from a patient's perspective (i.e., achieving optimal procedural comfort and minimizing procedural stress and failure) [18, 19]. Drugs traditionally used for PS (e.g., chloral hydrate, midazolam, barbiturates, and lytic cocktails) are associated with a substantial risk of procedural failure, discomfort, extended sedation times, and deeper sedation levels than intended with associated safety risks [20, 21]. Patient comfort is currently considered a primary goal of procedural sedation [22]. It has been argued that young children who are anticipated to suffer from substantial emotional distress need a titrated form of PS, including deep sedation, in order to have a successfully completed procedure, and to avoid major psychological trauma to the child, the family, and healthcare staff [23, 24]. The application of forced immobilization by physical restraint is increasingly considered as inhumane and unacceptable in nonlifesaving procedures [22, 25].

We searched the literature for available evidence on essential professional skills and competence required for effective and safe PS in children. Results were used to define evidence-based recommendations on the skills and competence a professional entrusted with PS should minimally possess, in order to be able to perform PS in children safely and effectively.

## 2. Methods

Literature was searched and selected by a multidisciplinary panel of the Dutch Institute for Health Care Improvement CBO, involved in the development of an evidence-based guideline on PS using the Evidence-Based Guideline Development (EBRO) methodology. Systematic searches were done in Medline, Cochrane Library, and Embase, using the Medical Subject Heading (MESH) search terms “Conscious sedation/all”, “Moderate sedation/all”, and “Deep sedation/all” and the free search-terms “sedation”, “pediatric sedation” and “procedural sedation”, in title or abstract. The search was limited to papers published between 1993 and March 2009, in 4 languages (Dutch, English, French, and German) and to human subjects aged 0–18 years. Results were systematically and repeatedly combined with the MESH-term “Drug Toxicity” and the MESH-terms of drugs, drug combinations, and drug groups available for PS (chloral hydrate, (lytic) cocktails, promethazine, chlorpromazine, pentobarbital, thiopental, midazolam, fentanyl, meperidine, ketamine, propofol, dexmedetomidine, remifentanil, nitrous oxide, opioids, benzodiazepines, antihistaminic, antipsychotics, barbiturates, nitrous oxide, and anesthetics). For all drugs, specific searches were done using the MESH subheading “*adverse effects*”. Additional combined searches were done using search terms for safety, effectiveness, and non-pharmacologic methods (hypnosis, distraction techniques, and play therapy). Textbooks and reference tables were systematically searched for additional papers.

Before inclusion in the pool of studies to be reviewed, all papers obtained were analyzed by the multidisciplinary panel for relevance and accuracy of definitions of safety and effectiveness.

In accordance with the EBRO methodology, selected papers were classified according to their methodological quality and strength of evidence: A1: systematic review including at least two independent A2-level studies, A2: randomized, double blinded comparative clinical trial of good quality and substantial size, B: comparative study, including retrospective cohort study and case-controlled trial, but not having all characteristics of an A2 study, C: noncomparative studies, and D: expert opinion. Findings from literature were summarized in conclusions. These conclusions were classified in 4 levels. Level 1: conclusion based on one A1 study or on at least two independent A2 studies, level 2: conclusion based on one A2 study or on at least two independent B studies, level 3: conclusion based on one B or C study, and level 4: conclusion based on expert opinion only. Finally, on the basis of the conclusions and remaining considerations (nonclassified) recommendations were formulated.

## 3. Results

### 3.1. Requisite Skills and Competence to Guarantee Optimal Safety

Many studies were found claiming the safety of all kinds of PS drugs in a variety of settings but usually in a limited series of patients. However, given an estimated incidence of severe adverse events of about 1/10000, the majority of these studies are insufficiently powered to prove such conclusion [18]. Most studies use vague definitions for the adverse reactions they report and consider the absence of directly life-threatening events as synonym for “safe”. In more recent observational studies on PS, the study setting is usually a strictly controlled, well-equipped, well-trained, and dedicated sedation team, which may differ appreciably from common settings in many practices around the world. Finally, well-designed controlled prospective studies in nonanesthesiologists analyzing the relationship between the level of professional skills/competence and the safety of PS are nonexistent. Therefore, evidence on this subject must be gathered in an indirect way. To do so, the following rationale was followed. At first, published critical analysis of PS-related incidents might elucidate the requisite competence and skills for PS. Next, the level of skills and competence professionals must achieve with regard to safety are likely to be determined by (1) the probability that a medicine may have undesirable adverse effects which require specific recognition and treatment, and (2) the predictability of the depth and duration of sedation of a medicine. The latter is important since unexpected deep sedation is associated with a higher rate of adverse events [21]. Out of all retrieved studies reporting PS-related adverse events, only those were selected for this systematic paper that reported the incidence of adverse events in large numbers of patients (>±1000), or that had studied adverse events following the use by nonanesthesiologists of the anesthetics propofol, ketamine, dexmedetomidine, remifentanil, and nitrous oxide. 

#### 3.1.1. Retrospective Critical Incident Analysis of PS-Related Adverse Events and Outcomes

In 2000, Coté published, in two separate papers, a retrospective critical incident analysis of adverse sedation events in pediatrics, as reported to the American Food and Drug Administration between 1969 and 1996. 95 incidents were reported, 51 resulting in death, 9 in permanent neurological injury, and 21 in prolonged hospitalization. Significant contributing factors were: “out of hospital” locations, inappropriate monitoring of physiological parameters, inadequate resuscitation skills, inadequate presedation medical evaluation, and inadequate recovery procedures. No particular medication was associated with a higher risk, except that overdosing and drug interactions (particularly when 3 or more drugs were used) were associated with mortality [4, 5]. Although the safety profile and the margins of safety vary among drugs, Coté showed that PS-related safety is determined by circumstances and professional skills, rather than by specific pharmacological characteristics. Professionals who do not have the requisite competence to recognize and treat the potential PS-related complications constitute a significant risk factor for the occurrence of fatal complications or complications causing permanent harm to the patient (*Level 3 conclusion* [4, 5]). 

#### 3.1.2. Reported Data on PS-Related Adverse Events

The studies stated below are summarized in Tables 1 and 2.

**Adverse Effects of Commonly Used Nontitratable Sedatives**

A retrospective study by Sanborn et al. of 16467 sedations during imaging procedures in children using chloral hydrate, midazolam, fentanyl, or pentobarbital found 70 (0.4%) respiratory incidents: desaturation only (*N* = 58), aspiration (*N* = 2), and airway obstruction requiring airway intervention (*N* = 10). The main risk factors were an underlying respiratory problem and the use of more than one sedative [27].

A prospective study by Malviya et al. in 1140 children, of which the majority were sedated with chloral hydrate for diagnostic imaging, showed a 5.5% incidence of respiratory complications leading to oxygen saturation of <90%: respiratory depression (4.7%), airway obstruction (0.6%), and apnea (0.17%). The risk of complications was significantly greater for more seriously ill children and for children less than one year old [28].

A risk analysis by Hofmann et al. based on prospectively collected data of 950 sedations using chloral hydrate, midazolam, fentanyl, pentobarbital, ketamine, or cocktails of 3 or more agents, identified 27 sessions (2.8%) in which a serious adverse event occurred: deep desaturation (*N* = 9), airway obstructions (*N* = 5), apneas (*N* = 3), aspirations (*N* = 2), hypotension, bradycardia (*N* = 2), or excessively deep or prolonged sedations (*N* = 6). Significant risk factors were the absence of a systematic risk assessment, a failure to follow safety guidelines, deep sedation, the simultaneous use of multiple agents, and the use of chloral hydrate [13].

A prospective international multicentre study of 30037 sedations by specifically trained professionals working in dedicated PS teams reported low incidences of major adverse events: desaturation (SatO2 < 90%) 1.57%, stridor 0.04%, laryngospasm 0.04%, apnea 0.24%, excessive airway secretions 0.41%, and vomiting 0.47%. The attending professional could adequately treat all complications. Cardiopulmonary resuscitation was necessary in one case. Anesthesiologists (19%), emergency physicians (28%), and intensivists (28%) administered the sedations. The most frequently used sedatives were propofol (50%), midazolam (27%), ketamine (14%), chloral hydrate (12%), pentobarbital (13%), and opiates (10%) [36].

Mason et al. reported in three separate comparative studies the adverse events of oral and intravenous (IV) pentobarbital used for PS in diagnostic imaging or nuclear medicine. Potentially severe adverse events like oxygen desaturation occurred extremely rare (<1%). Compared to oral chloral hydrate and intravenous pentobarbital, oral pentobarbital is associated with significantly less desaturations (resp., 1.6% versus 0.2% and 0.9% versus 0.2%) [35, 38, 39].

A retrospective study by Roback et al. in 2500 successive children undergoing PS in an emergency department (ED) showed that the incidence of respiratory complications depended on the medication used: 5.8% for midazolam, 6.1% for ketamine, 10% for ketamine + midazolam, and 19.3% for midazolam + fentanyl [33].

A prospective study by Newman et al. of 1341 PS sessions in children in an ED showed an incidence of serious complications of 11.9% (96,2% hypoxia, 1,3% hypotension, and 2,5% stridor). 92% of the complications occurred during the actual procedure, whereas the rest occurred after the procedure up to 40 minutes after the last dose of sedative. The risk of complications depended strongly on the medication used: midazolam 1.4%, ketamine + midazolam + atropine 9.8%, and midazolam + fentanyl 21.5% [34].

Another ED study by Pena and Krauss of 1180 successive children, using intravenous medicines (midazolam + fentanyl *N* = 391, midazolam *N* = 67, fentanyl *N* = 21, ketamine *N* = 40, pentobarbital *N* = 93, lorazepam *N* = 9, or midazolam + morphine *N* = 1), intramuscular ketamine (*N* = 180), oral medicines (midazolam *N* = 62, ketamine *N* = 2, chloral hydrate *N* = 122, diazepam *N* = 1, and lorazepam *N* = 1), rectal chloral hydrate *N* = 4), intranasal medicines (midazolam *N* = 3, midazolam + sufentanil *N* = 25), and nitrous oxide (*N* = 168), showed an overall complication incidence of 2.3% (*N* = 27). The following complications occurred: desaturation < 90% requiring intervention (*N* = 10), apnea (*N* = 1), larynx spasm (*N* = 1), bradycardia (*N* = 1), stridor with vomiting (*N* = 1), and 1 child started to vomit while being ventilated with a mask/bag applied to treat desaturation. There was no significant difference in the incidence of adverse events between the different sedation medicines [37].

A prospective study by Pitetti et al. of 1244 sedations in 1215 children in an ED showed an incidence of adverse events of 17.8% including desaturation (*N* = 178), stridor (*N* = 6), hypotension (*N* = 2), vomiting (*N* = 4), a rash (*N* = 7), agitation (*N* = 9), hiccups (*N* = 3), and dizziness (*N* = 3). An antidote had to be administered 6 times (3 flumazenil, 3 naloxone) and 2 patients sedated with fentanyl + midazolam required respiratory interventions (one with a Mayo cannula and one with mask/bag ventilation). The risk of complications depended heavily on the medication used. Patients sedated with midazolam + fentanyl had a significantly higher risk of adverse events (161/686 = 23.4%), compared to patients who had been treated with midazolam + ketamine + atropine (24/277 = 8.6%) or IV midazolam (1/65 = 1.5%) [32].

In a randomized controlled trial by Yildizdas et al. of 126 children undergoing a PS for painful oncology procedures, patients were randomly assigned for one of five forms of intravenous PS: ketamine (1 mg/kg), midazolam (0.15 mg/kg), ketamine + midazolam (1 mg/kg + 0.1 mg/kg), midazolam + fentanyl (0.1 mg/kg + 2 micrograms/kg), and propofol (2 mg/kg). Patients were monitored through saturation measurement and capnography. Patients sedated with midazolam + fentanyl and with propofol had a significantly more desaturations and hypercapnia compared to the three other groups. Desaturations were observed in 0%, 0%, 8%, 28%, and 52%, respectively, whereas hypercapnia was found in 0%, 0%, 0%, 4%, and 12%, respectively [8].

**Adverse Effects of Propofol**

Barbi et al.'s prospective study concerned deep PS with propofol administered by nonanesthesiologists (1059 procedures in 827 children aged 0–21 years old: gastroscopies (*N* = 483), colonoscopies (*N* = 289), and painful procedures (*N* = 173). All sedating professionals had followed a specific training, including theoretical and practical training on propofol, airway management, mask/bag ventilation, and resuscitation. Of the 1059 patients, 34 (12.6%) had a transient desaturation that resolved spontaneously. Deep desaturation with the need for mask/bag ventilation was required in 4/483 patients (0.8%) undergoing a gastroscopy, in 1/287 patients (0.3%) undergoing a painful intervention, and in 0/289 patients (0.0%) undergoing a colonoscopy. Laryngospasm occurred in 10/483 patients (2.1%) who underwent a gastroscopy. In 24 of the 483 gastroscopies (4.9%), an anesthesiologist was urgently required. In 13/24 cases (54.2%), this concerned assistance with the laryngoscopic insertion of an endoscope, in 10/24 cases (41.7%), the treatment of a laryngospasm and in 1/24 cases (4.2%), assistance to deal with a serious esophageal bleed. The trained professionals were able to manage adequately all adverse events that occurred during colonoscopies and painful interventions [12]. Propofol for PSA in children administered by specifically trained nonanesthesiologists has also been studied in pediatric oncology, radiology, and emergency medicine. A retrospective study by Hertzog et al. found that in 251 propofol sedations by pediatric intensivists hypotension (50%) and respiratory depression requiring transient bag-valve-mask ventilation (6%) were the most important adverse events [40]. A prospective study by the same authors in 28 oncology patients, undergoing 50 sedations, showed similar results: transient hypotension (64%) and partial airway obstruction (12%) were the most important adverse events. Apnea requiring bag-valve-mask ventilation occurred in 2% of procedures [41]. In a retrospective case series by Pershad and Godambe (*N* = 52) of propofol PS in the ED, no patient required assisted ventilation or developed clinically significant hypotension. The incidence of respiratory depression requiring airway repositioning or supplemental oxygen was 5.8% [42].

Bassett et al. analyzed prospectively 293 propofol sedations in children on an ED. Transient decrease in systolic blood pressure without clinical signs of poor perfusion was found in 92% of the patients. Nineteen patients (5%) had hypoxia, 11 patients (3%) required airway repositioning or jaw-thrust maneuvers, and 3 patients (0.8%) required bag-valve-mask ventilation. No patient required endotracheal intubation [43]. In a similar study by the same authors in 87 patients (291 sedation sessions), partial airway obstruction requiring brief jaw-thrust maneuver was noted for 4% of patient sedations. Transient apnea requiring bag-valve-mask ventilation occurred in 1% of patient sedations [44].

Vespasiano et al. reported a prospective study on 7304 propofol sedations outside the operation room in 4464 children, undergoing MRI (42.8%), non-MRI diagnostic imaging (22.5%), hematology/oncology procedures (26.2%), or other procedures (10.5%). All sedations were performed by pediatric intensivists according a sedation program that was in adherence to the American Academy of Pediatrics guidelines. The program was locally governed by a multidisciplinary committee with representation from anesthesiology, critical care, nursing, oncology, cardiology, and emergency medicine. To assess the overall safety profile of propofol a specific quality audit tool was designed. Hypotension (>25 mmHg drop from baseline) occurred in 31.4% of the patients but was mostly without circulatory compromise. High-volume fluid therapy was necessary in only 0.11% of cases. Infrequent respiratory adverse events were laryngospasm (0.27%), regurgitation without aspiration (0.05%), regurgitation with aspiration (0.01%), and bronchospasm (0.15%). Almost 5% of patients had an oxygen desaturation (1.73% between 85–90%; 2.9% < 85%) while airway obstruction requiring an oral or nasal airway occurred in 2% of cases. Unfortunately, ETCO_2_ was not evaluated systematically in this study. Only 0.37% of the patients needed bag-valve-mask ventilation because of hypopnea and/or apnea. All side effects could be managed successfully by the sedation team. There were no cardiac arrests. Patients with an abnormal airway (as defined by an airway score) were significantly more likely to develop oxygen desaturation or airway obstruction. None of the intended procedures or sedations had to be aborted [29].

The multicentric Pediatric Sedation Research Consortium (PSRC) collected prospectively data on 49836 propofol sedations in children. The PSRC consists of anesthesiologists, pediatric medical subspecialists, emergency physicians, pediatric intensivists, nurses, physician assistants, and health care research personnel who seek to continuously improve the quality, safety, effectiveness, and cost of pediatric sedation/anesthesia practice. Participants work in 37 different locations, including large children's hospitals, children's hospitals within hospitals and general/community hospitals. Following an initial study, the group meeting in this consortium agreed on a collective mission statement regarding pediatric procedural sedation. Decisions were based on guidelines from the American Academy of Pediatrics, American Society of Anesthesiologists, and American College of Emergency Physicians regarding sedation/anesthesia of pediatric patients, a review of the literature and the consensus of the consortium members. Besides sharing a common mission on PS, the PSRC is a data-sharing group: all participators agree to perform periodic audits of records to assure data and to maintain a prospective registry of all patients receiving PS [36].

Transient O_2_ desaturation below 90% for more than 30 seconds occurred 154 times per 10000 propofol administrations (1.5%). Central apnea or airway obstruction occurred 575 times per 10000 administrations (5.8%). Per 10000 encounters stridor occurred 50 times (0.5%), laryngospasm 96 times (0.96%), excessive secretions 341 times (3.4%), and vomiting 49 times (0.49%). Aspiration occurred 4 times during these 10000 sedation/anesthesia encounters (0.04%). There were no deaths. Cardiopulmonary resuscitation was required twice (0.02%). The sedating professionals could manage all adverse events appropriately. In an unadjusted analysis, the rate of pulmonary adverse events was not different for anesthesiologists versus other providers. Young age (<6 months), fasting time <8 hours, ASA classification III or higher and concomitant use of opoids were all significantly related with a higher risk for respiratory adverse events [26].

**Adverse Effects of Ketamine**

Several authors studied ketamine for PS during oncology procedures (lumbar punctures, bone marrow punctures, and/or bone biopsies), performed by non-anaesthesiologists. Evans et al. reported an incidence of desaturation of 1.7% and no airway obstruction during 119 sedation sessions [46]. Cheuk et al. reported an incidence of desaturations of 8.7% during 369 sedation sessions. These desaturations only required brief treatment with oxygen. No apneas or airway obstructions occurred [48]. In a prospective study by Meyer et al. of 183 PS sessions, potentially serious complications were desaturation <90% (5.4%) and laryngospasm (0.5%) [47]. Both intravenously (IV) and intramuscularly (IM) administered Ketamine were studied for PS in painful ED procedures. In a recent meta-analysis of 8282 children receiving PS with ketamine for procedures in an ED, the overall incidence of respiratory adverse events was 3.9%. Independent risk factors were high intravenous doses, administration to children younger than 2 years or aged 13 years or older, and the concomitant use of anticholinergics or benzodiazepines. Variables without independent association included oropharyngeal procedures, underlying physical illness (American Society of Anesthesiologists class > or = 3), and the choice of intravenous versus intramuscular route [30]. A retrospective analysis by Green et al. of a series of cases (*N* = 636) in which sedation with ketamine was administered by pediatric gastroenterologists for gastrocopies in children, showed a high incidence of laryngospasm (13.9% in the age group <6 years; 3.6% in the age group >6 years) [45].

**Adverse Effects of Dexmedetomidine**

In the last few years, dexmedetomidine has been studied for PS in children undergoing painless procedures. Regarding effectiveness for sedation in diagnostic imaging dexmedetomidine is significantly superior to midazolam and similar to propofol [49, 50]. Berkenbosch et al. published a prospective case series reporting the use of Dexmedetomidine in 48 children. Heart rate, blood pressure, and respiratory rate decreased but remained within normal limits for age. End-tidal CO_2_ exceeded 50 mm Hg in seven of 404 measurements (1.7%) [53]. Mason et al. studied dexmedetomidine for sedation for computer tomography imaging (CT) in 62 patients. Heart rate (HR) and mean arterial blood pressure decreased an average of 15% and no significant respiratory changes were observed [54]. In another study (*N* = 250), these authors showed that individual titration of dexmedetomidine for CT imaging is associated with modest fluctuations in HR and blood pressure which were independent of age, required no pharmacologic interventions, and did not result in any adverse events [51]. In a prospective study by the same group, dexmedetomidine as sole agent for pediatric MRI was studied in 747 consecutive patients. Three different dosing groups were analysed. Bradycardia without hypotension occurred in 16% of cases. There were no respiratory adverse events [52]. Ray and Tobias retrospectively reviewed the charts of 42 children with autism pervasive developmental disorders and epilepsy, who received dexmedetomidine for sedation during electroencephalography. No significant hemodynamic or respiratory effects were noted [55]. In two separate randomized controlled trials, Koroglu et al. compared dexmedetomidine with respectively midazolam and propofol for sedation in children undergoing MRI scanning. No relevant adverse events were seen in the children sedated with dexmedetomidine (*N* = 70) [49, 50].

**Adverse Effects of Remifentanil**

Remifentanil, a potent ultrashort acting synthetic opioid, has been studied for PS in children undergoing short painful procedures (e.g., lumbar puncture, and bone marrow puncture), both as a sole agent and combined with Midazolam or Propofol. Litman reported a high incidence of potentially life threatening respiratory depression in children undergoing painful procedures with the combination of a benzodiazepine and remifentanil. Out of 31 patients 25 (80.6%) developed an apnea, requiring constant stimulation, and 10 (32.3%) became hypoxemic [58] Keidan et al. published a randomized controlled trial comparing propofol (*N* = 36) and propofol-remifentanil (*N* = 41) for bone marrow aspiration in children. The addition of remifentanil was associated with a decrease in propofol dose and, consequently, recovery time, but with an increased risk of respiratory depression: hypoventilation or hypoxemia were significantly more frequent if remifentanil was added (19.5% versus 11.1%) [56] In a recent randomized controlled trial by Antmen et al.(A2), eighty children undergoing bone marrow aspiration were randomly assigned to one of four sedation regimens: remifentanil 1 mcg/kg (*N* = 20), midazolam 0.05 mg/kg + remifentanil 0.5 mcg/kg/min (*N* = 20), alfentanil 20 mcg/kg (*N* = 20), and midazolam 0.05 mg/kg + alfentanil 20 mcg/kg (*N* = 20). Relevant adverse events occurred in none of the 4 groups [62].

**Adverse Effects of Nitrous Oxide**

A French multicentric prospective study by Gall et al. of 7,511 sedation sessions with 50% nitrous oxide/50% oxygen premix, investigated the incidence of serious complications (oxygen desaturation, airway obstruction, apnea, bradycardia and/or oversedation). Such complications occurred in 25 sessions (0.3%). In all cases, the problems dissolved instantly after discontinuation of the administration of nitrous oxide, without any need for airway intervention or ventilation. The main risk factors were age (<1 year) and the simultaneous administration of benzodiazepines and opiates [31].

Zier et al. reported a case series of 1018 sedation sessions using nurse-administered nitrous oxide (continuous flow; concentration of 70%) for urinary catheterization. Only minor adverse events (diaphoresis, nausea, and vomiting) were observed in 4% of the sessions. Oversedation without respiratory compromise occurred in 0.8% of cases [61]. Babl et al. studied prospectively the relationship between fasting status and adverse events in 220 patients receiving nitrous oxide in a pediatric ED. Fasting status was obtained in 218 patients (99.1%). Of these, 155 (71.1%) did not meet fasting guidelines for solids. There were no serious adverse events and no episodes of aspiration. Emesis occurred in 7% of cases. There was no significant difference in median fasting time between patients with and without emesis [59].

The same author studied prospectively the safety of high-concentration continuous-flow nitrous oxide (70% versus 50%) in children (*N* = 762, age range 1–17 yrs). Sixty-three (8.3%) patients sustained mild and self-resolving adverse events, most of which were vomiting (5.7%); 2 patients (0.2%) had serious adverse events. Both serious events (1 chest pain and 1 desaturation) occurred in the group of 70% nitrous oxide. There was no significant difference in adverse events rates between nitrous oxide 70% (8.4%) and nitrous oxide 50% (9.9%) [60].

#### 3.1.3. Data from Literature on the Predictability and Controllability of Sedation Depth

It has been shown that unexpected deep sedation is associated with a higher rate of adverse events [13, 21]. The predictability of final sedation levels of a certain drug therefore determines the skills and competence the professionals in charge should possess. A search was made of existing literature on this subject. Results are summarized in Table 3.

Motas et al. published an observational study in 86 children who underwent PS using midazolam, midazolam in combination with fentanyl or pethidine, chloral hydrate, pentobarbital, or ketamine. Sedation depth was assessed by an independent observer, using a validated sedation scale and by bispectral cerebral function monitoring (BIS). These observations were compared to the sedation depth the practitioners set out to achieve. The intended sedation depth was reached in 72% (sedation scale) and in 52% (BIS) of the cases, respectively. In 35% of the cases, the BIS figure present was consistent with general anesthesia. The incidence of airway complications was significantly higher in the group that had been deeply sedated unintentionally [21].

A risk assessment by Hoffman et al. based on prospective collected data of 96 sedations for widely varying procedures with chloral hydrate (15%), midazolam (28%), fentanyl (1%), pentobarbital (28%), ketamine (2.8%), or cocktails of 3 or more of the medicines (5.7%), showed that in 22% of the procedures, a deep sedation level was reached, although deep sedation had only been intended in 7% of the procedures [13].

Malviya et al. studied two different types of discharge criteria in 29 children who had been sedated for an echocardiography (27/29 = 93.1% with chloral hydrate and 2/29 = 6.9% with midazolam + diphenhydramine). Standard criteria (normal vital parameters, normal oxygen saturation, return to original consciousness level, normal cough and swallowing reflexes, and normal movement) were compared with an objective assessment of the consciousness using BIS monitoring and two validated scales of observation. The objective criteria correlated better with being fully awake than the standard criteria but it took significantly more time before those objective criteria were reached [63].

The under 1.2 cited study by Newman et al. (prospective study of 1341 PS sessions in children in an emergency department (ED)) showed an incidence of serious complications of 11.9% of which 92% occurred during the actual procedure, whereas the rest occurred after the procedure up to 40 minutes after the last administered dose of sedative [34].

### 3.2. Requisite Skills and Competences to Guarantee Optimal Effectiveness

Effectiveness is named as an outcome measure in most of the studies on PS published over the last few decades. Mutual comparisons or combining averages is impossible, because the definition of effectiveness varies considerably for each procedure, or because it is not properly defined at all. No prospective controlled studies were found comparing different levels of professional skills/competence and the effectiveness of PS. Evidence was therefore searched in an indirect way by looking for which PS techniques a professional should master in order to achieve optimal PS effectiveness. We defined that *an optimal PS technique should achieve near 100% predictable procedural success and timing, an optimal match between desired and achieved levels of sedation, minimal induction and recovery times, and an optimal patient comfort by minimizing procedural pain, anxiety and the need for physical immobilization or restraint*. Next, we looked for settings and techniques with published evidence for contributing in reaching this optimal level. Results were classified as conclusions in four different categories of techniques or strategies with a proven effect on PS effectiveness. 

#### 3.2.1. Effect of the Introduction of a Dedicated Well-Trained Team for PS on the Effectiveness of PS

Several authors have shown that the introduction of a dedicated PS team that works according to published guidelines results in a significant decrease of procedural failure (*Level 2 conclusion* based on Hoffman et al. 2002(B), Ruess et al. 2002(C), and Sury et al. 1999(C)) [13, 15, 64]. Although it is impossible to deduce from those studies to what extent this result is due to specific professional skills and competence, PS seems to become more effective when specifically trained professionals perform PS in accordance with international guidelines.

#### 3.2.2. The Superiority of Titratable Medicines or Medicines with a Highly Predictable Effectiveness, Including Deep Sedation

In order to achieve an optimal level of effectiveness, each PS should ideally be directed to an individually determined sedation level. This makes the use of short acting drugs (e.g., propofol) that can be titrated to the desired level of sedation (including deep sedation) advantageous over the use of long acting drugs. There is growing evidence for the need for deep sedation for the majority of procedures in pediatrics. A retrospective analysis by Dial et al. of the sedation depth that was eventually required for a category of examinations (*N* = 32) that were not (very) painful and for which immobility was not strictly required turned out to be *deep sedation* after all in 26/32 cases (81.3%). For the category of painful and invasive examinations for which local anesthesia was used, light to moderate sedation turned out to be sufficient in only 4/156 cases (2.6%), whereas deep sedation was necessary in 136/156 cases (87.2%) and even a general anesthesia in 16/156 cases (10.3%) [24]. On the other hand there is good evidence for the superior effectiveness of PS with titratable medicines with a clearly predictable effectiveness. This has been demonstrated in children undergoing very painful procedures (e.g., oncological procedures, procedures in an ED), (protracted) stressful procedures (e.g., endoscopies) and procedures for which patients need to lie still for long periods (e.g., for imaging and radiotherapy). In addition, working with propofol also leads to a significantly shorter induction time and a significantly quicker recovery. Having the requisite competencies and skills to use this sort of sedatives safely therefore seems important to guarantee optimal effectiveness (*Level 1 conclusion* based on Migita et al. 2006(A1), Marx et al. 1997(A2), Pershad et al. 2007(A2), Dalal et al. 2006(B), Seiler et al. 2001(B), Iannalfi et al. 2005(B), Kohsoo et al. 2003(B), and Holdsworth et al. 2003(B)) [65–71].

#### 3.2.3. Deployability of Techniques for Light Sedation

Children often have to be physically forced or restrained for so-called “minor painful procedures” (e.g., blood sampling, inserting an intravenous access, suturing a wound, lumbar puncture, bone marrow puncture, changing a dressing, incision of abscess, resection of naevus or cyst, bladder catheterization, intraarticular injection, and Ear Nose Throat procedures). It has been demonstrated that the level of comfort during such interventions can be considerably improved when nitrous oxide is used. Nitrous oxide in concentrations of up to 70%, when combined with nonpharmacological distraction techniques and adequate topical anesthesia, is a very effective and safe way to suppress procedural pain and stress in children >1 year old. (*Level 3 conclusion* based on Iannalfi et al. 2005(B), Kanagasundaram et al. 2001(C), Burnweit et al. 2004(C), Frampton et al. 2003(C), and Zier et al. 2007(C) [61, 72–75]. For children undergoing reduction of an uncomplicated forearm reduction nitrous oxide in concentrations of 50% in combination with local anesthetics is equally effective as intravenous ketamine but is associated with a significantly shorter recovery time and less respiratory side effects. (*Level 3 conclusion* based on Luhmann et al. 2006(B)) [76]. In children that need to receive sutures, nitrous oxide in concentrations of 50% in combination with local anesthetics controls the procedural pain and stress more effectively than orally taken midazolam or local anesthetics alone (*Level 2 conclusion* based on Luhmann et al. 2001(B) and Bar-Meir et al. 2006(B)) [77, 78]. Inserting a venous access in children who are known to have difficult veins is easier under sedation with nitrous oxide + topical anesthesia than topical anesthesia alone. (Level 3; Ekbom et al. 2005(B)) [79]. Topical anesthesia and nitrous oxide combined are more effective than topical anesthesia or nitrous oxide alone. (*Level 1 conclusion* based on Paut et al. 2001(A2) and Hee et al. 2003(A2)) [80, 81]. 

#### 3.2.4. Use of Nonpharmacological Techniques

In literature, good evidence is available for the importance of applying nonpharmacological techniques to improve procedural success and comfort. When a professional takes care over providing good information about the procedure to be followed, this may result in the children feeling less stress during the procedure and being less scared about future procedures (*Level 3 conclusion* based on Claar et al. 2002(C) and Bishop et al. 2002(C)) [82, 83]. Adequate information also helps parents to provide better support for their children during a painful procedure (*Level 3 conclusion* based on Kupietzky and Ram 2002(C) and Cline et al. 2006(D)) [84, 85]. A child (>4 years old) that receives sufficient preparation (e.g., by information, practice, simulation, and play therapy) before an MRI examination, a gastroscopy or nuclear examination will experience less distress during the procedure and will require less sedation or analgesia (*Level 2 conclusion* based on Mahajan et al. 1998(A2), Rosenberg et al. 1997(B), Presdee et al. 1997(C), Awogbemi et al. 2005(C), and de Amorim e Silva et al. 2006(C)) [86–90]. Between 1993 and 2009 3 high-quality Systematic Reviews (SR) of nonpharmacological interventions for procedure-related pain in children have been published, allowing 3 *Level 1 conclusions*. Cepeda et al. (2006; A1 including 51 RCT's of which only 4 addressed procedural pain in children) could not demonstrate evidence for the effectiveness of music therapy during intravenous cannulation and vaccination in children. Although listening to music reduced pain intensity scales in general and opioid requirements in particular, the reported effects are small. Pooling of the 4 studies was impossible due to different quantification methods of pain intensity [91]. Richardson et al. published a SR (2006; A1 including 7 RCTs and 1 non-RCT) on the pain reducing effects of hypnosis in pediatric cancer patients undergoing common painful procedures (infusapost access, venipuncture, lumbar puncture, and bone marrow aspiration). Although 7/8 studies included reported a significant reduction of pain, the authors conclude that due to methodological limitations there is no conclusive evidence for a significant effect of hypnosis on procedure-related pain [92]. Finally, the SR by Uman et al. (2006; A1 including 28 trials) showed a significant effect of distraction and hypnosis on self-reported pain during needle-related procedures (intramuscular injection, vaccination, venipuncture, intravenous cannulation, lumbar punction, and bone marrow aspiration). For other psychologhical techniques no significant effect on procedure-related pain could be concluded [93].

An additional SR by Kleiber and Harper (1999) focused on the effects of distraction on self-reported pain in children during intravenous cannulation, lumbar punction, bone marrow aspiration, injection, venipuncture, dental procedures, and burn treatment. They showed that distraction causes a significant reduction of self-reported pain. An important limitation of this SR is the fact that no details are provided on the methodological quality of the included studies [94]. None of the SR could demonstrate any adverse events of nonpharmacological techniques. 

Hypnosis on children reduces procedure-related pain and distress more effectively compared to *local anesthesia* (venipuncture and lumbar puncture; *Level 1 conclusion* based on three independent A2 studies by the same authors: Liossi et al. 2009, Liossi et al. 2006, and Liossi and Hatira 2003), to *cognitive behavioral therapy* or *no therapy* (bone marrow aspiration; *Level 2 conclusion* based on Liossi and Hatira 1999(A2)), and to *standard medical care* including relaxation exercises or play intervention (cystogram; *Level 2 conclusion* based on Butler 2005(A2)) [95–99].

In conclusion, we found that a professional able to use psychological techniques for distraction or hypnosis during painful and/or stressful medical procedures may be able to reduce the child's procedural distress. Furthermore, the use of psychological techniques intended to distract children during a painful and/or stressful medical procedure reduces the need for sedation (*Level 2 conclusion* based on Harned and Strain 2001(B) and Train et al. 2006(B)) [100, 101].

## 4. Discussion

This paper shows sufficient evidence to support the statement that safety and effectiveness of PS are significantly related to the level of professional skills and competence. Although there are no prospective studies comparing the effect of different levels of skills and competence on PS related safety and effectiveness, this systematic paper identified in the relevant literature which competences and skills a professional should possess or achieve in order to be able to perform PS in children safely and effectively. For that purpose, we systematically summarized the results in conclusions classified according to the strength of evidence of the contributing papers. These conclusions can be translated into recommendations on the general skills and competence any professional entrusted with PS must have in order to achieve optimal safety and effectiveness (Tables 4 and 5). 

Besides general recommendations, we formulated additional recommendations depending on the level of sedation. Contrary to the generally accepted division between mild, moderate, and deep sedation in most guidelines, we believe that, based on the evidence, having different levels of monitoring and competence for moderate and deep sedation is arbitrary and potentially dangerous. Ever since the first guideline on PS was published, authors have linked the level of sedation with potential respiratory and cardiovascular side effects and by this with necessary safety precautions, monitoring, and professional skills and competence [10]. Consequently, definitions were made for *mild sedation*, *moderate sedation* (formerly called “conscious sedation”), *deep sedation,* and *anesthesia*. Mild sedation, formerly called “anxiolysis”, is typically the result of one standard dose of midazolam or by the breathing of nitrous oxide (inspired concentration up to 50%) [60]. Higher doses, or other drugs, either alone or in combination, are likely to cause deeper levels of sedation. Commonly used PS drugs intended for moderate sedation such as chloral hydrate, barbiturates, benzodiazepines with/without opioids, and solely opioids cause wide variations in depth of sedation. If a single dose is given the goal of moderate sedation is not achieved or exceeded in a substantial number of children. Therefore, for individual cases, prediction of the effective sedation end point is unreliable [21]. Multiple doses or combinations of drugs are more likely to cause deep sedation and are associated with hypoventilation, respiratory depression, and serious morbidity. Considering sedation levels as a sliding scale, rather than a step-by-step change in consciousness, the transition from one level to another can be subtle and sudden. It is, therefore, advisable to recommend the same safety precautions and professional skills for all levels of sedation beyond mild sedation, irrespective of the drug used for PS. Consequently, it is wise to formulate separate recommendations regarding professional skills and competence for mild sedation on one hand and for moderate to deep sedation on the other hand (Tables 5(a) and 5(b)). Although the safety profiles of PS drugs are clearly different, the likelihood that potentially serious adverse events may happen and the predictability of depth and duration of sedation are clearly more important. Both issues have a direct impact on the imperative skills and competence, mainly in terms of timely recognition and appropriate management of possible adverse events. PS-related safety is determined by logistics, organization and professional skills rather than by specific pharmacologic characteristics.

In order to achieve an optimal level of effectiveness, each PS should ideally be directed to an individually determined sedation level. This makes the use of short-acting “titratable” drugs advantageous over the use of long-acting drugs. Short-acting drugs can be used to overcome the pain and distress that varies according to the procedures and the patients themselves. It can be concluded from this systematic paper that professionals having the requisite skills and competence to work with titratable anesthetics (e.g., propofol) are able to achieve more optimally an effective PS for children undergoing very painful procedures (e.g., oncological procedures, procedures in an ED), (protracted) stressful procedures (e.g., endoscopies) and procedures for which patients need to lie still for long periods (e.g., diagnostic imaging and radiotherapy). In particular, young children (<6 years) are in need of deep sedation sometimes even for so called “mild” procedures [23, 24]. Although the obvious advantages of titratable deep sedation (e.g., using propofol) over other sedatives for many procedures in children are increasingly emphasized in recent literature, the term *deep sedation* has been under discussion, because it may be indistinguishable from general anesthesia. While this point may be overstated it has led to the widespread recommendation that the same personnel, equipment, and facilities must be available to manage both deep sedation and anesthesia. The most important severe adverse effect of propofol is respiratory depression, which is associated with unexpected deep sedation and can arise suddenly and unexpectedly [1]. As a consequence the question whether nonanesthesiologists can be safely entrusted with the use of this potent drug has been a matter of debate [23]. There is an obvious reluctance by the anesthetic world to entrust trained non-anesthesiologist with highly active anesthetic drugs [23, 102]. However, in many countries, a clear trend is seen to entrust deep sedation to specifically trained non-anesthesia professionals in particular because of the scarcity of anesthesiologists. Emergency physicians, intensivists and gastroenterologists have been prominent in this development [12, 29, 40, 41, 102, 103]. In addition, It has been shown that in optimal safety and monitoring conditions deep sedation using propofol is equally safe irrespective whether it is administered by trained nonanesthesiologists or anesthesiologists [26, 102]. An evidence-based clinical practice advisory for the administration of propofol for PS by nonanesthesiologists was recently published [104].

For minor painful procedures the deployability of short-acting mild sedation using nitrous oxide and ability to apply adequate topical anesthesia are essential skills for optimal effectiveness. In addition, not only the ability to define and apply an individually tailored PS technique but also the ability to implement nonpharmacological techniques, such as distraction, hypnosis and combined cognitive-behavioral interventions, belongs to the essential competence and skills. 

Finally, we found evidence that the application of published guidelines within a well organized, well trained, and dedicated PS team will enhance PS related safety and effectiveness.

In conclusion, PS has to be considered as a separate medical act, provided by well-trained, competent, and skilled professionals only, working within a context of transparency, registration, and ongoing quality control. Skills and competence, rather than professional title, are determinants for safe and effective PS. We believe that these evidence-based recommendations regarding necessary skills and competence should be used to set up training programs and to define which professionals can and cannot be credentialed for PS in children. Much emphasis is needed for adequate and effective implementation strategies for these recommendations.

## Figures and Tables

**Table 1:** Overall conclusions regarding the relation between professional competence/skills and PS-related safety.

Nr	Conclusion	Quality level
(1)	Serious PS related adverse events occur *more frequently *	
	(I) *In children with an underlying disease. *	Level 1
	(A1) Green et al. 2009 [30]	
	(B) Sanborn et al. 2005 [27], Cravero et al. 2009 [26]	
	(C) Malviya et al. 1997 [28], Vespasiano et al. 2007 [29]	
	(II) *If multiple sedatives are used *	Level 1
	(A1) Green et al. 2009 [30]	
	(B) Hoffman et al. 2002 [13], Pitetti et al. 2003 [32], Sanborn et al. 2005 [27], Cravero et al. 2009 [26]	
	(C) Gall et al. 2001 [31]	
	(III) *In young children *	Level 1
	(A1) Green et al. 2009 (<2 years) [30]	
	(B) Cravero et al. 2009 (<6 months) [26]	
	(C) Malviya et al. 1997 (<1 year) [28], Gall et al. 2001 (<1 year) [31]	
	(IV) *In certain drugs compared to others*:	
	(IV.1) The combination of a benzodiazepine with an opiate (e.g., midazolam + fentanyl) is associated with a	Level 2
	higher risk of respiratory complications (21–23%) compared to the use of midazolam alone or ketamine	
	with midazolam.	
	(A2) Yildizdas et al. 2004 [8]	
	(B) Pitetti et al. 2003 [32], Roback et al. 2005 [33], Newman et al. 2003 [34]	
	(IV.2) Oral pentobarbital is associated with less adverse events compared to oral chloral hydrate	Level 3
	(B) Mason et al. 2004 [35]	
	(IV.3) In comparison with ketamine, midazolam and ketamine + midazolam, midazolam + fentanyl and	Level 2
	propofol generate a higher risk of hypoventilation and desaturation.	
	(A2) Yildizdas et al. 2004 [8]	

(2)	Serious PS-related adverse events occur *less frequently* if specifically trained professionals working in dedicated	Level 2
	teams perform sedation according to international guidelines.	
	(B) Barbi et al. 2003 [12], Hoffman et al. 2002 [13], Cravero et al. 2009 [26]	
	(C) Vespasiano et al. 2007 [29]	

**Table 2:** Drug-specific conclusions regarding the relation between professional competence/skills and PS-related safety.

Nr	Conclusion	Quality Level
*Nontitratable drugs intended for moderate to deep sedation*		

(1)	During PS, intended to moderate or deep sedation, with the use of benzodiazepines, chloral hydrate, barbiturates, opiates, or combinations of these medicines, and during the subsequent recovery phase, there exists a variable but real risk of potentially serious drug-induced adverse events. Especially the risk for respiratory depression and/or airway obstruction necessitates specific skills and competence from the professionals in charge in terms of recognition and treatment.	Level 2
	(B) Hoffman et al. 2002 [13], Pitetti et al. 2003 [32], Sanborn et al. 2005 [27], Cravero et al. 2006 [36],	
	Roback et al. 2005 [33], Newman et al. 2003 [34], Pena et al. 1999 [37], Mason et al. 2001 [38],	
	Mason et al. 2004 [35], Mason et al. 2004 [39]	
	(C) Malviya et al. 1997 [28]	

*Propofol*		

(1)	During PS using propofol, there is a real risk of potentially serious drug-induced adverse events. Especially the risk for respiratory depression and/or airway obstruction necessitates specific skills and competence from the professionals in charge in terms of recognition and treatment.	Level 3
	(B) Cravero et al. 2009 [26]	
	(c) Barbi et al. 2003 [12], Hertzog et al. 1999 [40], Hertzog et al. 2000 [41], Pershad and Godambe 2004	
	[42], Bassett et al. 2003 [43], Guenther et al. 2003 [44], Vespasiano et al. 2007 [29]	

(2)	PS with propofol, including deep sedation, is equally safe in the hands of anesthesiologists and nonanesthesiologists if the latter are well trained and part of dedicated sedation team.	Level 3
	(B) Cravero et al. 2009 [26]	
	(C) Barbi et al. 2003 [12], Vespasiano et al. 2007 [29]	

(3)	A deep PS using ketamine or propofol for examination of the upper airways, or for endoscopies of the upper gastrointestinal system, carries a real risk of potentially serious complications (i.e., laryngospasm and deep desaturation), which require specific skills and competence from the professionals in charge in terms of recognition and treatment.	Level 3
	(C) Barbi et al. 2003 [12], Green et al. 2001 [45]	

*Ketamine*		

(1)	During PS using ketamine, there is a small but real risk of potentially serious drug-induced adverse events. Especially the risk for respiratory depression, airway obstruction and—infrequently—laryngeal spasm necessitates specific skills and competence from the professionals in charge in terms of recognition and treatment.	Level 1
	(A1) Green et al. 2009 [30]	
	(C) Green et al. 2001 [45], Evans et al. 2005 [46], Meyer et al. 2003 [47], Cheuk et al. 2005 [48],	

(2)	Independent risk factors for respiratory adverse events during a PS with the use of ketamine are high intravenous doses, administration to children younger than 2 years or aged 13 years or older, and the coadministration of anticholinergics or benzodiazepines.	Level 1
	(A1) Green et al. 2009 [30]	

*Dexmedetomidine*		

(1)	Based on a limited published experience on the use of dexmedetomidine for PS by experienced professionals, there seems to be a very small risk of potentially serious drug-induced adverse events. Respiratory events are extremely rare and hemodynamic adverse events (i.e., bradycardia and hypotension) are mostly clinically insignificant. Specific experience in dosing techniques, individual titration and avoiding dexmedetomidine in those patients who may not tolerate hemodynamic fluctuations seems to be associated with low risks.	Level 1
	(A2) Koroglu et al. 2005 [49], Koroglu et al. 2006 [50]	
	(B) Mason et al. 2008 [51], Mason et al. 2008 [52]	
	(C) Berkenbosch et al. 2005 [53], Mason et al. 2006 [54], Ray and Tobias 2008 [55]	

*Remifentanil*		

(2)	During PS using remifentanil, there is a real risk of potentially serious drug-induced adverse events. Especially the risk for respiratory depression and/or airway obstruction necessitates specific skills and competence from the professionals in charge in terms of recognition and treatment.	Level 2
	(A2) Keidan et al. 2001 [56]	
	(C) Litman 1999 [57], Litman 2000 [58]	
*Nitrous Oxide*		

(1)	PS with nitrous oxide is associated with an extremely low chance of serious adverse events. Instant discontinuation of gas flow in case of respiratory depression is the most important rescue intervention.	Level 2
	(B) Babl et al. 2005 [59], Babl et al. 2008 [60]	
	(C) Gall et al. 2001 [31]	

(2)	Specific risks for adverse events during nitrous oxide administration are:	Level 3
	(I) A young age (<1 year old)	
	(C) Gall et al. 2001 [31]	
	II. Simultaneous use of other sedatives	
	(C) Gall et al. 2001 [31]	

(3)	In patients sedated with nitrous oxide, there exists no significant difference in median fasting time between patients with and without emesis	Level 3
	(B) Babl et al. 2005 [59]	

(4)	Nitrous oxide 70% causes significantly deeper sedation compared to nitrous oxide 50%. However, if embedded in a comprehensive sedation program there exists no significant difference in adverse events rates between both regimens.	Level 3
	(B) Babl et al. 2008 [60]	
	(C) Zier et al. 2007 [61]	

**Table 3:** Conclusions regarding predictability and controllability of nontitratbale drugs intended for PS.

Nr	Conclusion	Quality Level
(1)	For a PS with medicines that are difficult to titrate and/or long-acting (e.g., chloral hydrate, midazolam, barbiturates, opiates or combinations), the eventual depth of sedation, effectiveness and duration of the sedation and timing of adverse events cannot reliably be predicted. Therefore, possible adverse effects of any possible sedation depth should always be anticipated in terms of recognition and treatment.	Level 2
	(B) Hoffman et al. 2002 [13], Newman et al. 2003 [34], Malviya et al. 2004 [63], Motas et al. 2004 [21]	

**Table 4:** General recommendations on necessary skills and competence for achieving optimal PS related safety and effectiveness in children.

Nr	Recommendations
(1)	Knowledge of the drug dosing, dosing techniques, indications, contraindications, and requisite precautions of the sedation technique used, acquired through specific training or demonstrable relevant experience.
(2)	Regular personal experience of the applied medication or technique*.
(3)	Applying the form of sedation that is most appropriate for the procedure and the patient. This implicates the ability to guarantee the optimally effective sedation level in a predictable manner. An optimal PS technique should achieve near 100% predictable procedural success and timing, an optimal match between desired and achieved levels of sedation, minimal induction, and recovery times and an optimal patient comfort by minimizing procedural pain, anxiety, and the need for physical immobilization or restraint.
(4)	The ability to perform preprocedural screening and a systematic risk analysis.
(5)	The ability to inform the patient, parents or carers about the sedation technique, the effects, potential side effects, and possible alternatives. The information must be given in time and be appropriate for the comprehension level of the patient and parents/carers.
(6)	The ability to guarantee a child-centered approach within a general policy that favors children before, during and after the procedure.
(7)	The ability to apply, or arrange for complementary nonpharmacological techniques like preparation, distraction, combined cognitive-behavioral interventions, and hypnosis.
(8)	The ability to (a) apply effective local or topical anesthesia, if appropriate, and (b) to recognize and intervene with possible toxicity of local anesthetic agents.
(9)	Organizing the necessary monitoring and rescue facilities during and after the procedure for as long as the consciousness level is lowered.
(10)	The ability to organize a supervised recovery phase and to define the discharge criteria.
(11)	The ability to organize the prompt availability of a resuscitation team or a professional trained in Pediatric Life Support.
(12)	Supervising, registering, assessing and optimizing the quality of the sedation in terms of safety and effectiveness.

*It is impossible to derive from literature a more precise definition of “regular personal experience”. The authors believe that regular experience means a minimal of 50 PS sessions per year.

**Table:** (a) Recommended specific additional skills and competence for achieving optimal safety during *moderate and deep sedation* in children.

Nr	Recommendations
(1)	In order to guarantee optimal levels of safety and effectiveness during a PS involving (a possibility of) moderate-to-deep sedation, the PS must be carried out by a separate professional that is not involved in the actual procedure.

(2)	During a PS involving (a possibility of) moderate or deep sedation and during the subsequent recovery phase, a professional must be present with at least the following additional competence and skills:
	(1) The ability to assess and interpret the sedation depth.
	(2) The ability to guarantee the necessary monitoring of vital parameters, including capnography, and being able to appraise
	and interpret the monitored information.
	(3) Having acquired the necessary *knowledge during a specialist course* and by means of *refresher courses* and ability to *manage *
	the following techniques at APLS* level:
	(3.1) Techniques intended to guarantee an open airway, including skills to manage larynx spasm and to use Laryngeal
	Mask Airways.
	(3.2) Techniques to administer mask/bag ventilation.
	(3.3) The use of antagonists.
	(3.4) Heart massage techniques.

*APLS: Advanced Pediatric Life Support.

**Table:** (b) Specific additional skills and competence for achieving optimal safety during *mild sedation/anxiolysis* in children.

Nr	Recommendations
(1)	During a PS involving mild sedation and during the subsequent recovery phase, a professional must be present with the at least the following additional competence and skills:
	(1) The ability to assess and interpret the sedation depth.
	(2) The ability to maintain continuous verbal contact with the patient in the absence of any other form of monitoring.
	(3) Having acquired the necessary *knowledge through a specialist course* and by means of *refresher courses* and the ability to
	*manage* the following techniques at BLS* level:
	(3.1) Techniques intended to guarantee an open airway.
	(3.2) Techniques to administer mask/bag ventilation.

*BLS: Basic Life Support.
